# Obituary of Dr. Angelo Di George

**DOI:** 10.1186/1824-7288-36-22

**Published:** 2010-03-04

**Authors:** Luigi Tarani, Maria C Digilio, Bruno Dallapiccola, Donna M Mc Donald-McGinn, Bruno Marino

**Affiliations:** 1 Dipartimento di Pediatria, Sapienza Università di Roma, Roma, Italy; 2 Ospedale Pediatrico Bambino Gesù, Roma, Italy; 3 The Children Hospital of Philadelphia, University of Pennsylvania School of Medicine, Philadelphia, PA, USA

## Obituary

Dr. Angelo M. DiGeorge a world renowned physician and pediatric endocrinologist, died at age of 88 years, on October 11, 2009 of kidney failure at his home in Philadelphia.

Dr. DiGeorge first gained international recognition in the mid-1960's for his ground breaking discovery of a disorder characterized by congenital absence of the thymus and associated abnormalities. This birth defect in now-a-day referred to as DiGeorge syndrome or Velocardiofacial syndrome (Shrprintzen Syndrome) or chromosome 22q11.2 deletion syndrome, since the majority of affected individuals have a distinct part of the long arm of this chromosome missing. DiGeorge syndrome includes a pattern of more than 200 different defects, including hypoplastic thymus and parathyroid glands, conotruncal heart defects, and a characteristic facial appearance. Velocardiofacial syndrome is marked by the association of congenital conotruncal heart defects, cleft palate or velar insufficiency, facial anomalies and learning difficulties. It is now accepted that these two syndromes represent the different expression of a unique disorder manifesting at different stages of life. DiGeorge Syndrome is one of the most common genetic disorders known, occurring in about one every 4,000 livebirths. The DiGeorge's original 1965 paper reporting this anomaly has been quoted more than 500,000 times worldwide and the Google search yields more than 700,000 citations. During his long professional career, DiGeorge was one of the key figures who contributed to transform the Philadelphia St.Christopher's Hospital for Children from a small community hospital into a nationally prominent medical institution. DiGeorge has authored more than 230 medical papers, abstracts and text book chapters and he has been an invited guest lecturer around the world.

DiGeorge was the son of two Italian immigrants, Antonio and Emilia (Taraborelli). He was born in South Philadelphia on April 15, 1921. Angelo himself told us that his teacher at primary school changed his Italian surname DiGiorgio into the "american" DiGeorge. He graduated at the top of his class from South Philadelphia High School for Boys in 1939 and was awarded the competitive White Williams Scholarship at the Temple University where he graduated with distinction in chemistry in 1943. DiGeorge received his medical degree with Honors from Temple University School of Medicine in 1946, and completed his internship at Temple University Hospital. He then left Philadelphia from 1947 to 1949 to serve as Captain and Chief of the Medical Service for the U.S. Army 124^th ^Station Hospital in Linz, Austria. After returning to Philadelphia, Angelo met his future wife, Natalie Picarello, who was a Registered Nurse at Temple Hospital. He completed his pediatric residency at St. Christopher's Hospital for Children and did a post-doctoral fellowship in endocrinology at the Jefferson Medical College in 1954.

DiGeorge joined the Department of Pediatrics of Temple University School of Medicine in 1952. In 1967 he became a Professor of Pediatrics and an Emeritus Professor in 1991. Concurrently, he was also an attending physician at St. Christopher's where he became the Chief of Endocrinology and Metabolism (1961-1989), and the Director of the Pediatric Clinical research Center (1965-1982). He served on the Pediatric Endocrinology Subboard of the American Board of Pediatrics from 1987 until 1992. He was a founding member and past president of the Lawson-Wilkins Pediatric Endocrine Society and was the primary author of the Endocrinology Chapter for the Nelson Textbook of Pediatrics, known by pediatricians around the world as the "Green Bible" for more than 40 years.

**Figure 1:**
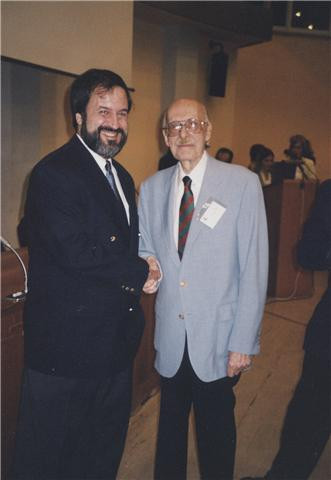
**Dr. Shprintzen, left, and Dr. Di George, right, met in Rome for the first time**.

On a personal level he was a compassionate physician who viewed the patient as a whole person, a superb diagnostician, a keen observer, a great teacher, a masterful lecturer, an absorbing storyteller, an avid reader, a literary writer and above all, a kind-hearted, fair-minded person. In addition to medicine, he had many other hobbies, including gardening, all of the performing arts, politics, stamp-collecting and Philadelphia sports, especially the Phillies. Dr. DiGeorge loved all things "Philly" and all things Italian. He first learned the art of debate on the debate team at South Philadelphia High School for Boys and he gladly engaged in animated debates on virtually any topic from sports to politics with his professional colleagues and at the family dinner table throughout his life. Dr Angelo DiGeorge was often invited to Italian scientific meetings, including the San Giovanni Rotondo Medical Genetic School and the Rome "Deletion 22q11" Meeting in 2002. It was in Rome that Angelo DiGeorge and Bob Shprintzen, the fathers of a unique disorder met for the first time (Fig. 1), although they were working on the same syndrome since a long time, living close to one another in USA. The contribute of Angelo DiGeorge to the history of Medicine is monumental and both patients and their families as well as doctors and scientists will be always grateful to him.

